# Study on the prognosis predictive model of COVID-19 patients based on CT radiomics

**DOI:** 10.1038/s41598-021-90991-0

**Published:** 2021-06-02

**Authors:** Dandan Wang, Chencui Huang, Siyu Bao, Tingting Fan, Zhongqi Sun, Yiqiao Wang, Huijie Jiang, Song Wang

**Affiliations:** 1 Department of Radiology, The Second Affiliated Hospital of Harbin Medical University, 246 Xuefu Road, Harbin, Heilongjiang Province China; 2 Department of Research Collaboration, R&D center, Beijing Deepwise & League of PHD Technology Co., Ltd, Beijing, China; 3 Department of Radiology, Longhua Hospital,, Shanghai University of Traditional Chinese Medicine, No.725, South Wanping Road, Shanghai, 200032 China

**Keywords:** Respiratory tract diseases, Outcomes research

## Abstract

Making timely assessments of disease progression in patients with COVID-19 could help offer the best personalized treatment. The purpose of this study was to explore an effective model to predict the outcome of patients with COVID-19. We retrospectively included 188 patients (124 in the training set and 64 in the test set) diagnosed with COVID-19. Patients were divided into aggravation and improvement groups according to the disease progression. Three kinds of models were established, including the radiomics, clinical, and combined model. Receiver operating characteristic curves, decision curves, and Delong’s test were used to evaluate and compare the models. Our analysis showed that all the established prediction models had good predictive performance in predicting the progress and outcome of COVID-19.

## Introduction

The emergence of the novel coronavirus, SARS-CoV-2, at the end of 2019 and the resulting Coronavirus Disease 2019 (COVID-19), rapidly spread throughout the globe in a short time, posing a great threat to human life and resulting in huge losses to the economy and the development of various countries. COVID-19 is characterized by symptoms of viral pneumonia, such as fever, fatigue, dry cough, and lymphocytopenia, quickly developing into acute respiratory distress syndrome, septic shock, refractory metabolic acidosis, bleeding, and coagulation disorders^1^. The severity of this disease and its prognosis are related to age, sex, with an increase in the mortality rate in patients with comorbidities, usually due to respiratory failure, shock, or multiple organ failure^2,3^. Supportive treatment to relieve symptoms and protect multiple organ functions is key to treating this disease. Timely identification and treatment of high-risk groups is key to reducing mortality and improving prognosis^4^.

The unique advantage of CT in the diagnosis of lung diseases makes it an important tool in diagnosing and treating patients with COVID-19. In a study comparing the diagnostic efficacy of chest CT and reverse transcription-polymerase chain reaction (RT-PCR) of patients with COVID-19, the sensitivity of CT diagnosis was 97% and the disease can be detected earlier than with nucleic acid test^5^. The most common CT-detectable manifestation of COVID-19 is predominantly peripheral ground-glass opacity (GGO) and consolidation. Abnormal chest CT can be seen even in asymptomatic patients, which rapidly evolves from unilateral localized to diffuse bilateral GGO within 1–3 weeks, and develops into consolidation or coexistence with consolidation. A combination of clinical and laboratory examination is helpful for the early diagnosis of COVID-19 pneumonia^6^. According to the recommendation of the Radiology Branch of the Chinese Medical Association (First Edition), the CT staging criteria of COVID-19 are divided into three periods, namely early stage, progressive stage, and severe stage, which are based mainly on the morphological changes judged by radiologists with the naked eye^7^. Nonetheless, the boundary between different imaging stages is blurred, the standard has not yet been unified, and the imaging stage does not fully correspond to the clinical stage, so it is impossible to make a timely and accurate judgment of the outcome and prognosis of the disease, thus increasing the difficulty of diagnosis and treatment.

Radiomics refers to the high-throughput extraction and conversion of large numbers of image features into data, which may reflect not only the macroscopic attributes of the disease, but also the microscopic attributes that cannot be easily obtained by doctors in the process of naked eye recognition, and can also be further combined with clinical, pathological, and genomics data to provide clinicians with more comprehensive data for clinical decision-making^8,9^. Thus, it has great prospects for the classification, differentiation, and prognosis judgement of diseases^10,11^. Several articles about radiomics and COVID-19 have been recently published. Fatemeh et al. found that radiomics from unenhanced chest CT images can accurately predict the extent and type of pulmonary opacities, as well as the patient outcome, by comparing the radiomics and clinical variables, with an AUC reaching 0.99 in predicting disease severity^12^. Li et al. constructed a model integrating information from radiomics and deep learning features to discriminate critical cases from severe cases using CT images, with an AUC up to 0.909^13^.

From the perspective of disease progress and outcome of patients with COVID-19, this study aims to explore the factors that affect the prognosis of patients and develop a predictive model, which is not only conducive to furthering our understanding of the disease, but also to provide significant clinical guidance for the management of patients, the rational allocation of medical resources, and the selection of appropriate treatment modalities.

## Methods

### Patients

The study was approved by the Ethics Committee of the Second Affiliated Hospital of Harbin Medical University (KY2019-183). The informed consent of patients was omitted with the permission of the Ethical Committee of the Second Affiliated Hospital of Harbin Medical University because the retrospective study design according to the ethical guidelines for Medical and Health Research Involving Human Subjects. All experiments were performed in accordance with relevant named guidelines and regulations. The inclusion criteria were as follows: (a) RT-PCR confirmed COVID-19 diagnosis; (b) chest CT imaging data were available, and; (c) baseline information could be obtained. The training cohort comprised 124 patients diagnosed with COVID-19 between January 25 and July 30, 2020. We reviewed the medical record system to obtain case data, including demographics, comorbidities, physiological data, laboratory tests, and CT images from the picture archiving and communication system (PACS). We chose the first laboratory examination and CT images during the patient’s hospitalization to conduct statistical analysis and modeling. The exclusion criteria were as follows: (a) substantial motion artifacts in the CT images (n = 15); (b) small or inconspicuous lesions that could not be identified by CT (n = 24); and (c) unavailable imaging or clinical data (n = 21). In total, 60 patients were excluded from the study.

The included patients were separated into two groups according to the course of the disease: aggravation and improvement. Aggravation was defined as a composite endpoint when patients developed respiratory failure, acute respiratory distress syndrome (ARDS), acute liver or kidney injury, or death. Similar methods have been used in previous studies to assess the severity of serious infectious diseases^14,15^. Patients lacking these conditions were placed in the “improvement” group. At the same time, another 64 cases from Provincial tuberculosis hospital were collected as an external validation cohort, called the test cohort, according to strict screening criteria mentioned earlier to minimize selective bias.

Of the 188 patients described in our study, 56 were included in another published work, which was a descriptive study focusing on the clinical characteristics of patients with or without entry into the Intensive Care Unit (ICU)^16^.

### CT imaging protocol

Image acquisition was performed using a Philips 256iCT and GE 11800i scanner. Patients were supine with head advanced, and continuous scanning was performed from the top of the lung to the bottom of the lung. Chest CT protocol was as follows: tube voltage, 120 kV; tube current, 200 mA; slice thickness, 5 mm; standard algorithm thin layer reconstruction to 1–2 mm. No patients received intravenous contrast medium.

### Image processing and lesion segmentation

**Figure 1:**
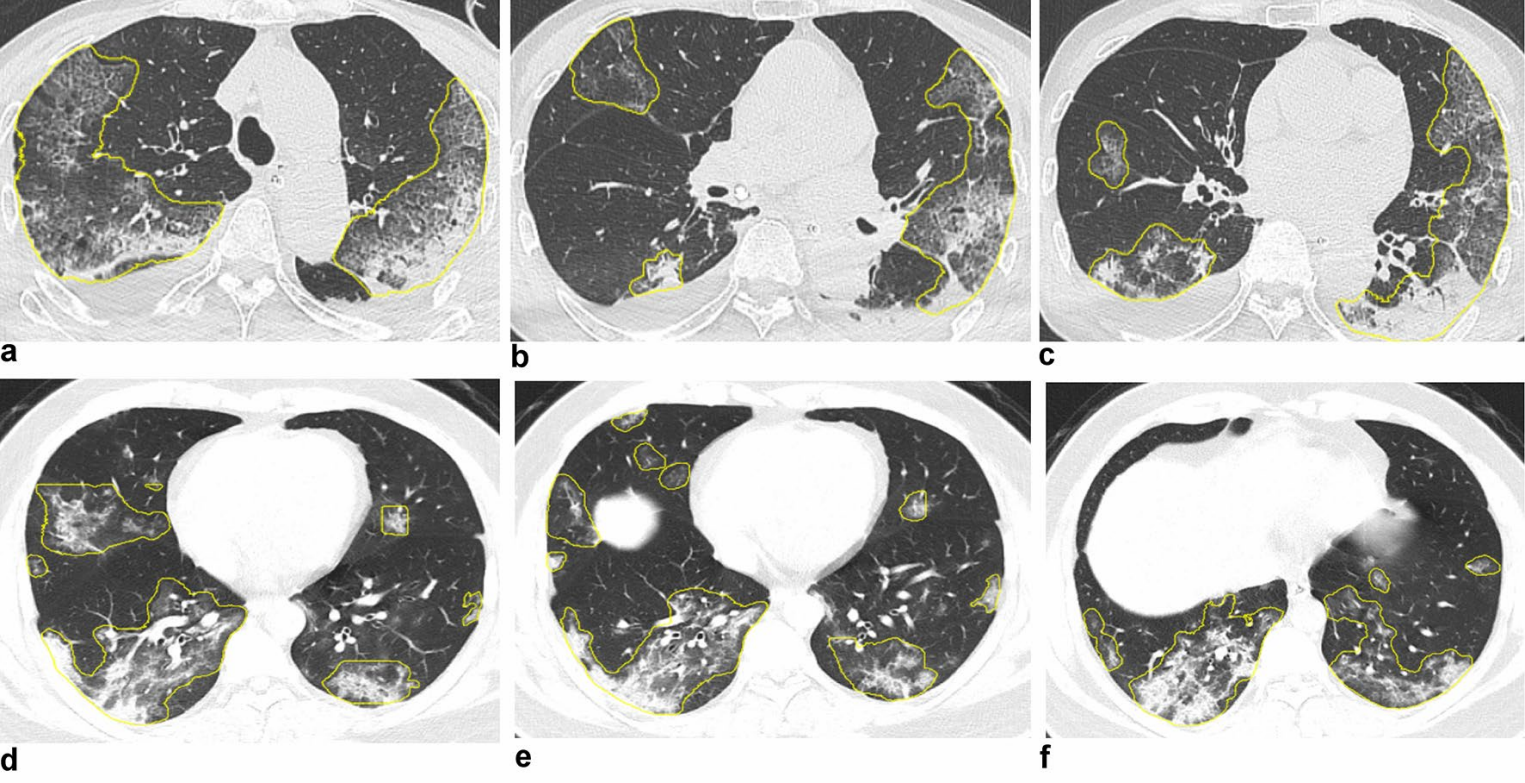
Schematic diagram of lesion segmentation. Examples of automatic recognition and segmentation of ROI of patients in the aggravation group and the improvement group, respectively. Both images show diffuse, multiple GGOs, and consolidation. (**a**–**c**) A 54-year-old male in the aggravation group; (**d**–**f**) A 32-year-old male in the improvement group.

Two radiologists with 3 and 7 years’ experience, respectively, in chest imaging diagnosis identified the lesions using the *Dr. Wise multimodal scientific research platform* (version number: V1.6, website: http://keyan.deepwise.com/) for automatic recognition and segmentation of signs of pneumonia. The region of interest (ROI) was manually examined and selected by doctors for feature extraction of typical viral pneumonia lesions, such as patchy GGO and interstitial changes in the early stage (especially in the periphery zone of the lung), multiple infiltrative GGO of both lungs or consolidation afterward, removed isolated small nodules below 3 mm, suspected simple bacterial infection, suspected old tuberculosis and suspected tumor lesions. Questionable lesions were confirmed by a third senior doctor. All the doctors were blinded to the patient’s outcome in this process. The schematic diagram of lesion segmentation is illustrated in Fig. 1.

### Feature extraction and modeling

Image standardization was performed using B-spline interpolation sampling technology for resampling, and all CT images were resampled to 1.0 × 1.0 × 1.0 mm^3^ voxels, which could effectively solve the influence of different machine parameters when scanning. The PyRadiomics package (Version 2.1.0, https://pyradiomics.readthedocs.io/) was used to extract image features from all labeled ROIs. The original CT image was pre-processed by wavelet transform and Laplacian Gaussian transform. Features were extracted from the pre-processed and original images.

Intra-class correlation coefficient (ICC) was calculated to evaluate the reliability and repeatability of observers. The first observer completed the segmentation of all lesions. After an interval of 14 days, 20 patients were randomly selected and segmented again to evaluate the repeatability within the observer. The second observer performed another segmentation of the above 20 patients, and the imaging features obtained by the first and second observers were compared to evaluate the repeatability between observers. The features with ICC ≥ 0.75 were considered to be reliable and stable to build models. Features were then screened by the F-test method. The radiomics models were established by fivefold cross-validation and five classical machine learning algorithms, namely Logistic Regression (LR), Support Vector Machine (SVM), Decision Tree (DT), Random Forest (RF), and Extreme Gradient Boosting (XGBoost).

**Figure 2:**
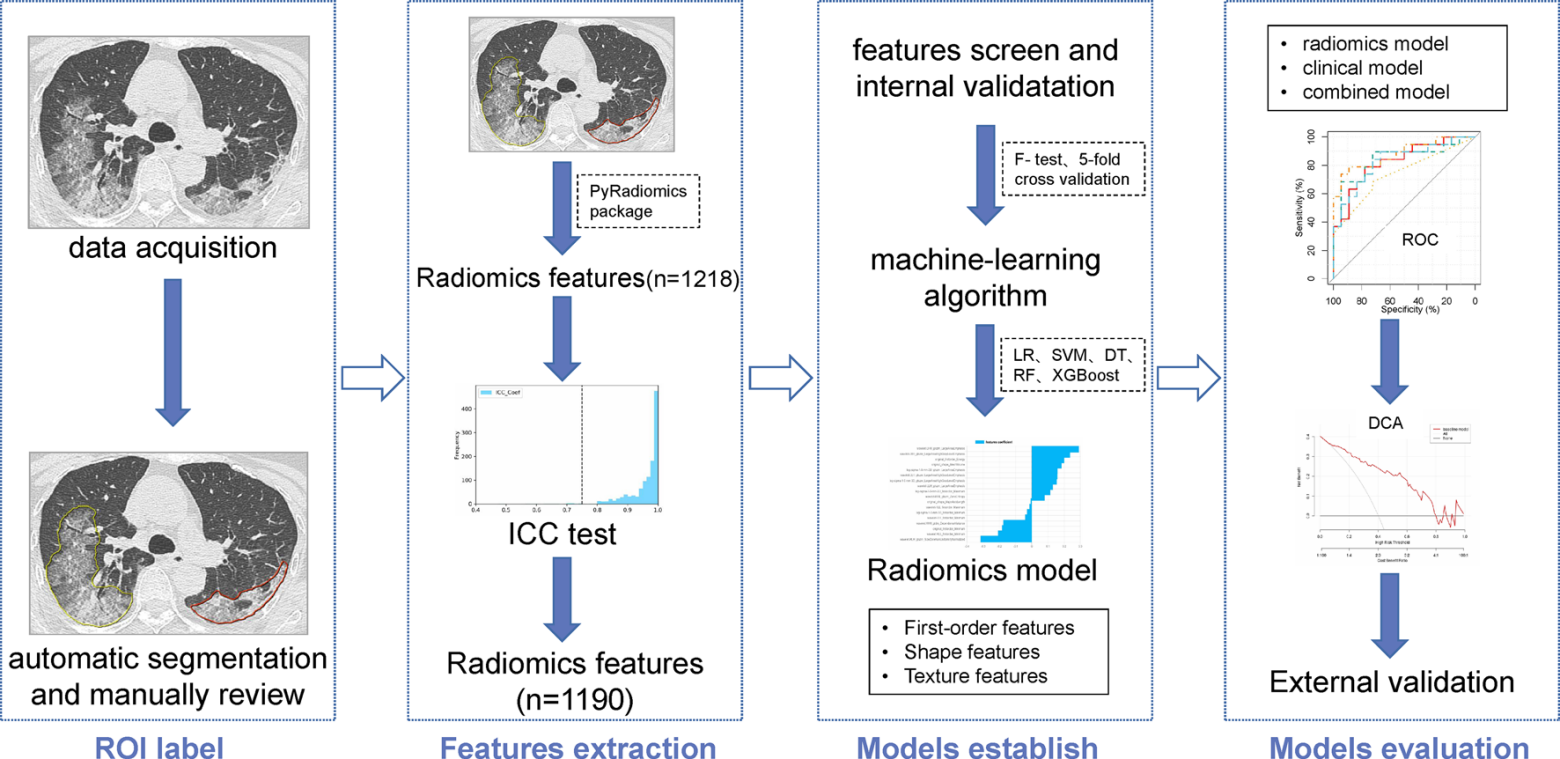
The workflow of the study.

Meanwhile, we constructed the clinical model with demographic and laboratory examination results, and the combined model integrating both clinical and radiomics models. The workflow of our study is shown in Fig. 2.

### Evaluation of model effectiveness

The effectiveness of the diagnostic model was evaluated by the area under the receiver operating characteristic (ROC) curve, namely AUC, sensitivity, specificity, and accuracy; the larger the AUC, the higher the prediction accuracy. The clinical application value of the model was evaluated using the decision curve analysis. External validation was conducted to evaluate the overfitting of the models.

### Statistical analysis

Software R (Version 3.6.3)^17^ was used for data analysis and figure plotting. For the analysis of measurement data, the Kolmogorov–Smirnov test was first used to evaluate normality, and the data with normal distribution and homogeneity of variance were analyzed by independent sample t-test and expressed as mean [standard deviation]. Otherwise, the data were analyzed by the Mann–Whitney U test and expressed as median [IQR]. Chi-square test and Fisher’s exact probability method were used to compare the differences between groups. DeLong’s test was used to compare the differences among the AUCs using the pROC package. Differences with *P* < 0.05 were considered to be statistically significant.

## Results

### Basic information

According to disease progression, 124 patients with COVID-19 in the training cohort were divided into two groups: 54 individuals in the aggravation group and 70 in the improvement group. Among them, there were 23 cases (18.54%) of respiratory failure, 15 cases (12.09%) of ARDS, 26 cases (20.96%) were complicated with acute liver or kidney injury, and 5 cases (4.03%) resulted in death. There were 102 cases with fever (82.3%), 78 cases with cough (63.4%), and 31 cases with muscle soreness (25%). The median time from hospitalization to composite endpoint was 8 days (range, 5–19 days). The statistical analysis revealed no significant differences in demographic information, such as age, sex, temperature, symptoms, and underlying diseases among patients. The test set included 64 patients, with 41 in improvement and 23 in aggravation group. The decreased lymphocyte contents, increased D-dimer, C-reactive protein and lactate dehydrogenase (LDH) showed a similar trend in *P*-value, and were selected to build the clinical model, together with age and sex, which are prognostic factors that couldn’t be ignored in clinic though with no statistical significance. Comparison of the basic information between the two cohorts is presented in Table 1. The demographic and laboratory characteristics are presented and compared in detail (see Supplementary Table S1 online).

**Table 1:** Comparison of baseline information in the training set and the test set.

	Training set (n = 124)	Test set (n = 64)	All (n = 188)	*P*-value
Age	57.0 [51.0;67.0]	58.5 [49.8;68.0]	57.5 [50.0;67.0]	0.887
**Sex**				0.437
Male	67 (54.0%)	30 (46.9%)	97 (51.6%)	
Female	57 (46.0%)	34 (53.1%)	91 (48.4%)	
Temperature (℃)	37.7 [37.0;38.2]	37.5 [36.8;38.1]	37.6 [36.9;38.2]	0.067
Length of hospitalization	21.0 [17.0;27.0]	19.0 [14.8;21.2]	20.0 [16.0;24.0]	0.001
Interval from symptoms to discharge	27.5 [23.0;35.2]	24.0 [21.0;28.0]	26.0 [22.0;32.0]	0.001
**Complication**				
Angiocardiopathy	33 (26.6%)	16 (25.0%)	49 (26.1%)	0.949
diabetes	26 (21.0%)	8 (12.5%)	34 (18.1%)	0.219
hypertension	36 (29.0%)	13 (20.3%)	49 (26.1%)	0.265
COPD	18 (14.5%)	9 (14.1%)	27 (14.4%)	1.000
chronic liver disease	10 (8.06%)	7 (10.9%)	17 (9.04%)	0.702
chronic kidney disease	8 (6.45%)	7 (10.9%)	15 (7.98%)	0.429
**Symptom**				
Fever	102 (82.3%)	42 (65.6%)	144 (76.6%)	0.018
Cough	78 (63.4%)	21 (32.8%)	99 (52.9%)	< 0.001
Muscular soreness	31 (25.0%)	8 (12.5%)	39 (20.7%)	0.070
Headache	16 (13.0%)	8 (12.5%)	24 (12.8%)	1.000
Diarrhea	15 (12.1%)	5 (7.81%)	20 (10.6%)	0.514
**Laboratory**				
white blood cell, *10^9^/L	5.32 [4.31;6.66]	5.38 [4.59;6.47]	5.33 [4.39;6.58]	0.750
Neutrophil, *10^9^/L	3.22 [2.52;4.58]	3.66 [3.09;4.60]	3.38 [2.65;4.58]	0.037
Lymphocyte, *10^9^/L	1.10 [0.69;1.45]	1.23 [0.90;1.44]	1.18 [0.72;1.45]	0.181
Hemoglobin, g/L	108 (21.5)	107 (17.8)	108 (20.2)	0.870
D-dimer, mg/L	1.85 [1.01;5.41]	1.90 [1.02;7.32]	1.88 [1.01;6.59]	0.258
C-reactive protein, ml/L	20.3 [8.78;52.0]	18.9 [10.2;27.1]	19.6 [8.78;39.8]	0.354
Albumin, g/L	37.3 [34.0;41.3]	36.7 [34.0;39.8]	37.1 [34.0;40.9]	0.330
LDH, U/L	428 [342;559]	399 [337;467]	411 [338;518]	0.055

Data are expressed as mean (standard deviation), median [IQR], or n (%), where n is the total number of patients in each group. p values are from independent sample t-test or Mann–Whitney U test (continuous variable), Chi-square test, or Fisher’s exact test (categorical variable). *P* < 0.05 indicates that it is statistically significant.

*LDH* lactate dehydrogenase, *IQR* interquartile range.

### ICC test results

In this study, a total of 1218 imaging features were extracted from the segmented ROIs in the training set. The ICC of only 28 features were below 0.75 and were removed, indicating that the automatic recognition function has good stability, and the established models are reliable. The bars of the intra and interobserver ICC is shown in Supplementary Fig. S1 online.

### Establishment and evaluation of the diagnostic models

**Figure 3:**
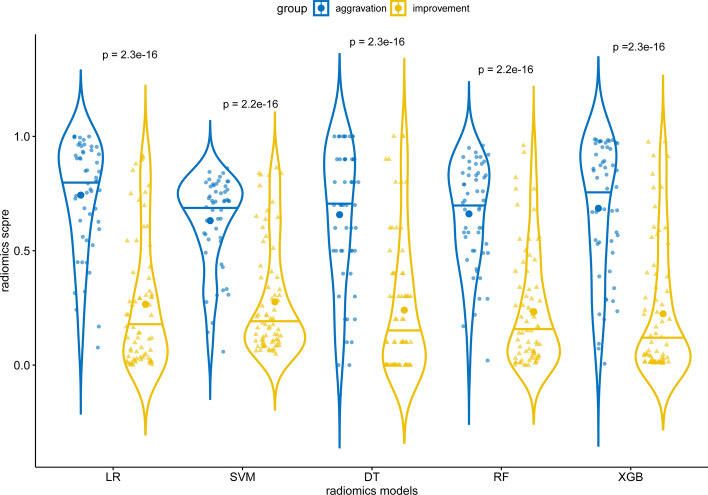
The violin plot of the radiomics scores between the aggravation and improvement groups of the five machine learning models. All the *p*-values are less than 0.05.

We used five machine learning classifiers (LR, SVM, DT, RF, and XGBoost) to establish the radiomics models. The radiomics score calculated by the regression coefficients of the final features multiplied by the value of the corresponding feature was significantly different in distinguishing the improvement and aggravation group in the five models (*p* < 0.05; Fig. 3). 19 features were eventually screened to establish the radiomics model, including 7 first-order features, 3 shape features, 9 texture features (1 gldm feature and 6 glszm features, 2 glrlm feature). Details of the selected features in the LR model are shown in Supplementary Table S2 and Fig. S2 online. Supplementary Figure S3 online shows the correlation heat map of the 19 selected features, with all the features’ correlation less than 0.9.

**Figure 4:**
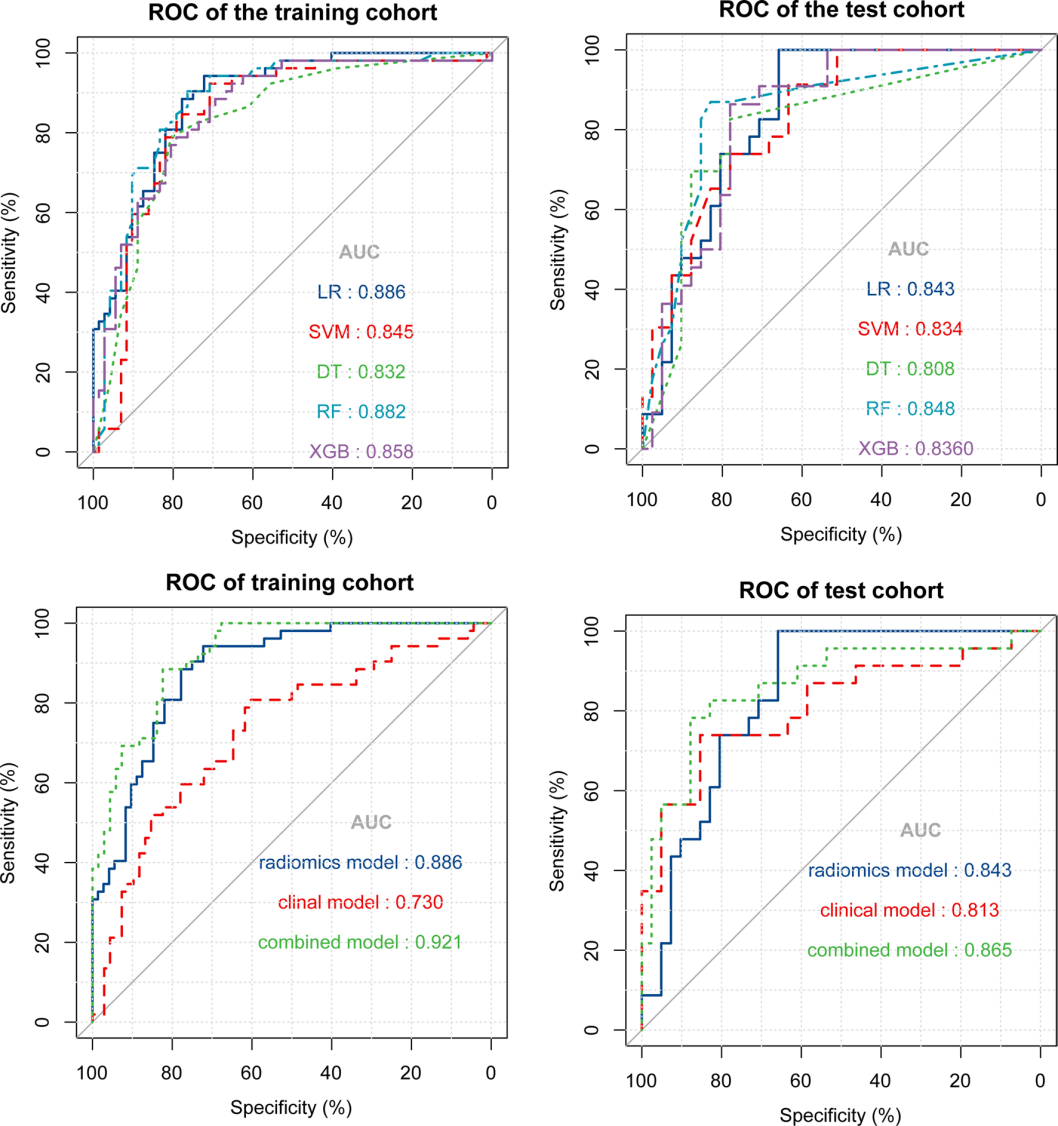
The ROC curves of the established models’ predictive performance in the training and test cohort. (**a**,**b**) ROC curves of the five radiomics models. (**c**,**d**) Comparison of ROC curves among the radiomics, clinical, and combined models.

The combined model was established by integrating the clinical and radiomics models. Prediction efficacy of the established models for disease progression was shown in Fig. 4. For the radiomics, clinical and combined models, the AUC values were 0.886 *vs* 0.730 *vs* 0.921 in the training set and 0.843 *vs* 0.813 *vs* 0.865 in the test set, respectively.

The statistical efficacy of the different models in the test set is presented in Table 2. The precision-recall graph of the LR radiomics model is shown in Supplementary Fig. S4 online. According to DeLong’s test, there were no significant differences among the radiomics, clinical and combined models in the test set as shown in Table S3. Predictive efficacy was also compared within the radiomics models (see Supplementary Table S4 online).

**Table 2:** Performance of the predictive models in the test cohort.

	Radiomics	Clinical	Combined
AUC [95% CI]	0.843 [0.731–0.922]	0.813 [0.696–0.900]	0.865 [0.757–0.938]
Best cut-off value	0.22	0.39	0.50
Sensitivity (%)	97.52	73.91	78.26
Specificity (%)	65.85	85.37	87.80
Negative predictive value (%)	98.45	85.37	87.80
Positive predictive value (%)	62.16	73.91	78.26
True positive rate (%)	99.12	73.91	78.26
False positive rate (%)	34.15	14.63	12.20
True negative rate (%)	65.85	85.37	87.80
False negative rate (%)	1.55	26.09	21.74
False discovery rate (%)	37.83	26.09	21.74
Accuracy (%)	78.13	81.25	84.38
Precision (%)	62.16	73.91	78.26
Youden Index J	0.6585	0.5928	0.6607
Recall	0.98	0.73	0.78
*P*-value	< 0.001	0.001	< 0.0001

*P* < 0.05 indicates that it is statistically significant.

*CI* confidence interval; *AUC* area under the curve.

**Figure 5:**
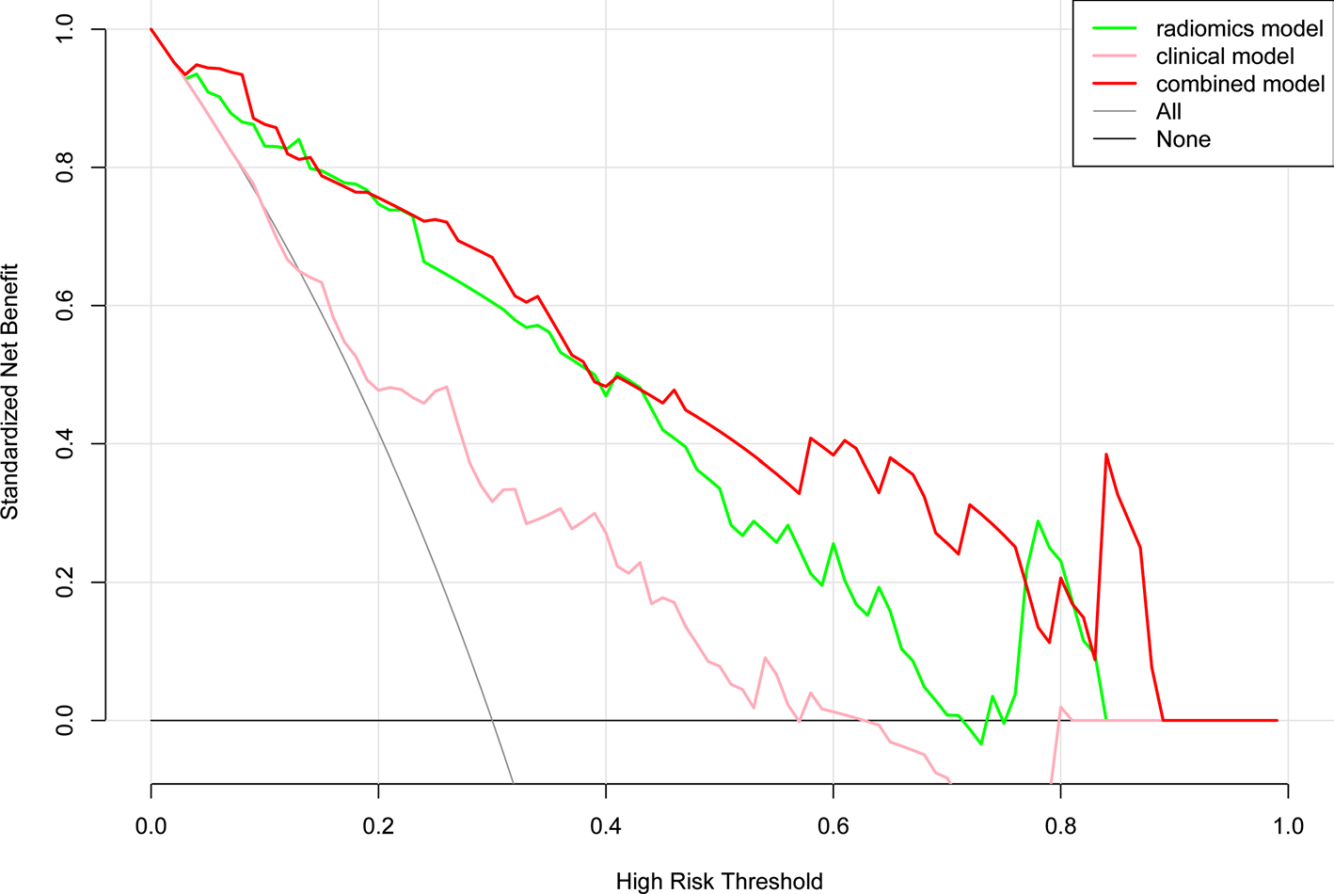
Decision curves of models. The horizontal axis represents the predicted risk range, ranging from 0.0 to 1.0, and the ordinate represents the net clinical benefit, calculated by summing the benefits (true-positive results) and subtracting the harms (false-positive results). The gray line is the net benefit of assuming that all patients were in the aggravation group; the black line is the net benefit of assuming no patients aggravated; the pink, green and red lines are the expected net benefit based on clinical, radiomics and combined model respectively, with the combined model (red line) showing the highest net benefit.

The overfitting evaluation of models in the external validation showed that there were no statistical differences in the AUC values between the training set and the test cohort (Table 3). The decision curves of the models showed great clinical application value^18^. The gray line is the net benefit of assuming that all patients were aggravated; the black line is the net benefit of assuming no patients aggravated; and the green, pink and red lines are the expected net benefit based on the predictive models, with the combined model (red line) showing the highest net benefit (Fig. 5).

**Table 3:** Overfitting evaluation of the prediction models.

Models	AUC [95%CI]		*P*-value
	Training cohort	Test cohort	
LR	0.886 [0.817–0.936]	0.845 [0.732–0.924]	0.4426
SVM	0.845 [0.769–0.904]	0.829 [0.713–0.912]	0.8556
DT	0.832 [0.754–0.893]	0.827 [0.711–0.911]	0.7253
RF	0.812 [0.769–0.904]	0.843 [0.729–0.922]	0.5767
XGBoost	0.858 [0.784–0.914]	0.836 [0721–0.917]	0.7146
Clinical model	0.730 [0.642–0.807]	0.805 [0.686–0.894]	0.2854
Combined model	0.921 [0.858–0.962]	0.865 [0.757–0.938]	0.3245

*P*-value reflected the differences between the training and test cohorts, and *P* < 0.05 (two-sided) were considered statistically significant.

*AUC* area under the curve; *CI* confidence interval; *LR* logistic regression; *SVM* support vector machine; *DT* decision tree; *RF* random forest; *XGBoost* extreme gradient boosting.

## Discussion

In this study, we comprehensively explored the clinical and radiomics characteristics of patients with COVID-19, focusing on establishing models in predicting the course of the disease. Results showed that using the first CT radiomics and clinical factors would play an ideal role to predict whether the disease would aggravate or improve, so as to make clinical decisions timely.

Due to advancements in imaging technology, diagnosis is developing from qualitative to quantitative, which can provide an objective evaluation of the heterogeneity of lesions^19^. Radiomics has been used in the field of oncology initially. Changes in voxels (reflected in the changes of radiomics features such as intensity, shape, texture or wavelet), can predict the response of a certain treatment^20^ and the survival time of the patient^21^; but there are only a few studies of radiomics in pneumonia. Rivka et al. had explored a radiomics model to predict pneumonitis induced by immunotherapy^22^. A review by Chumbita et al. highlighted recently published artificial intelligence approaches being used to support clinical decision-making processes in pneumonia^23^. With regard to COVID-19, Chen et al.^24^ constructed a radiomics nomogram based on CT images to predict disease prognosis. However, they divided patients into absorption group and consolidation (progression) group, based only on the condition of radiological progression, rather than the development of the patients’ actual clinical condition mentioned in our article. Furthermore, they included only 40 patients in the study and segmented 180 ROIs. They regarded each ROI as an independent case for grouping and analysis. In our study, we took the patient as a unit, not the lesion, which could minimize the overfitting phenomenon caused by different ROIs from the same patient. Chao et al.^25^ used holistic information containing imaging and clinical data for COVID-19 outcome prediction. Their grouping standard (endpoint) was the need for ICU admission. However, the decision on whether a patient required admission to the ICU is determined by many uncertain factors and is a relatively subjective judgement. Our endpoint is relatively objective, based on whether the patient has serious complications or death in practice.

The clinical and laboratory characteristics of COVID-19 patients also play an important role in predicting the development of the disease. A study of critically ill patients with COVID-19 from Yang et al.^26^ showed worse prognosis in older patients and, compared with survivors, non-survivors were older (64.6 vs 51.9 years old). Cho et al. reported that the mortality rate of men is higher, especially for patients aged 50–64 or ≥ 65 years^27^. In our study, there were no significant differences in demographic characteristics. The reasons for this discrepancy may be due to the small sample size. With regard to laboratory examination results, we observed a decrease in the number of lymphocytes and the content of hemoglobin, while the levels of C-reactive protein, D-dimer, and LDH were significantly increased. Elevated levels of C-reactive protein reflect an active inflammatory response in the body. According to a previous study, the increased D-dimer levels can increase the risk of thrombosis, embolism, and disseminated intravascular coagulation (DIC), and is strongly associated with disease progression and prognosis^28,29^. The level of LDH increased significantly in aggravated patients, and was related to organ injury. Other laboratory examination results were different between the two groups, but the values were either in the normal range or the differences were not significant in the test set, so they were not included in the clinical model. Although there was no statistical significance according to Delong’s test, the combined model showed better performance than the clinical or radiomics models alone. Therefore, in a clinical setting, we still need to make a comprehensive judgment by considering all aspects of the patient’s information, rather than just one aspect.

The results of our study can help medical personnel judge disease development promptly and take appropriate measures in the early stage, such as timely admission to the ICU for close monitoring, and early application of visceral protective drugs to avoid more serious consequences that may endanger people's lives. Further, our study may help guide the reasonable allocation of medical resources, providing a new method for the management of patients with COVID-19.

Our research has some limitations: Firstly, due to the short period for collecting case information, the patients were not followed up after discharge, and only the disease course and prognosis during hospitalization were discussed, which will be supplemented in the follow-up study. Secondly, because of the retrospective study design, there is a certain bias in the selection of cases. Thirdly, the overall number of cases is relatively small, and the sample size must be expanded and the models verified by multicenter studies in the future. Fourthly, this paper does not include the treatment of patients during hospitalization, and cannot rule out the impact of medical intervention on the progress of the disease, thus affecting the prognosis. Lastly, the fact that patients received no intravenous contrast media was another limitation, since the presence of acute pulmonary embolism may carry prognostic information.

In conclusion, the predictive models we established based on clinical and radiomics factors can effectively predict the development of COVID-19, by predicting whether the patient's condition will aggravate at the early stage of hospitalization, so that healthcare providers can take corresponding measures in advance and improve the efficiency of clinical decision-making.

## Supplementary Information

Supplementary Information.

## Data Availability

All data of the article can be obtained from the correspondent author by e-mail.
