# Supplementary material for: Study on the prognosis predictive model of COVID-19 patients based on CT radiomics
Source: Sci Rep. 2021 Jun 2;11:11591. doi: 10.1038/s41598-021-90991-0 (PMC8172890; doi:10.1038/s41598-021-90991-0)
Supplement: Supplementary file 1 — Supplementary Information. [file 41598_2021_90991_MOESM1_ESM.pdf]

Study on the prognosis predictive model of COVID-19 patients based on CT radiomics

Dandan Wang[1], Chencui Huang[2], Siyu Bao[2], Tingting Fan[1], Zhongqi Sun[1], Yiqiao Wang[1], Song Wang[3], and Huijie Jiang[1*]

[1]Department of Radiology, The Second Affiliated Hospital of Harbin Medical University, Harbin, China

[2]Department of Research Collaboration, R&D center, Beijing Deepwise & League of PHD Technology Co., Ltd, Beijing, China

[3] Department of Radiology, Longhua Hospital， Shanghai University of Traditional Chinese Medicine

*** corresponding author:**

Huijie Jiang, PhD, Department of Radiology, The Second Affiliated Hospital of Harbin Medical University, 246 Xuefu Road, Harbin, Heilongjiang Province, China.

E-mail: jianghuijie@hrbmu.edu.cn

Song Wang, PHD. Department of Radiology, Longhua Hospital，Shanghai University of Traditional Chinese Medicine, No.725, South Wanping Road, Shanghai, 200032, China.

E-mail: songwangws@163.com

Supplement Figures

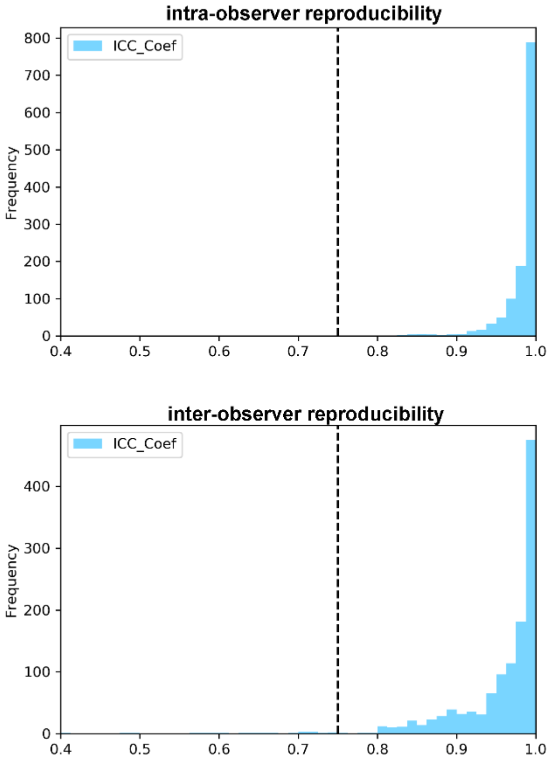

Figure S1. The bars of intra- and inter-observer's ICC. A coefficient greater than 0.75 is considered to have a good consistency. 28 coefficients were less than 0.75, that is, the parts to the left side of the dotted line in the bottom panel. ICC_Coef: intra-class correlation coefficient.

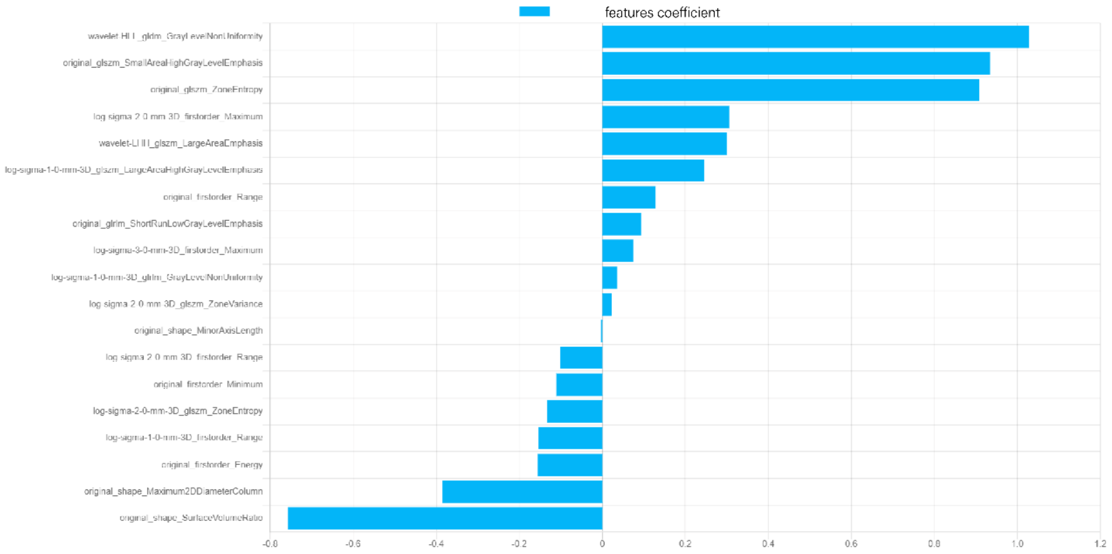

Figure S2. The bar diagram of the selected features with different relative weights in the logistic regression model. The features' coefficient less than 0 is a negative correlation, on the contrary, it is a positive correlation. The larger the absolute value of the feature coefficient is, the higher the relative weight in the model.

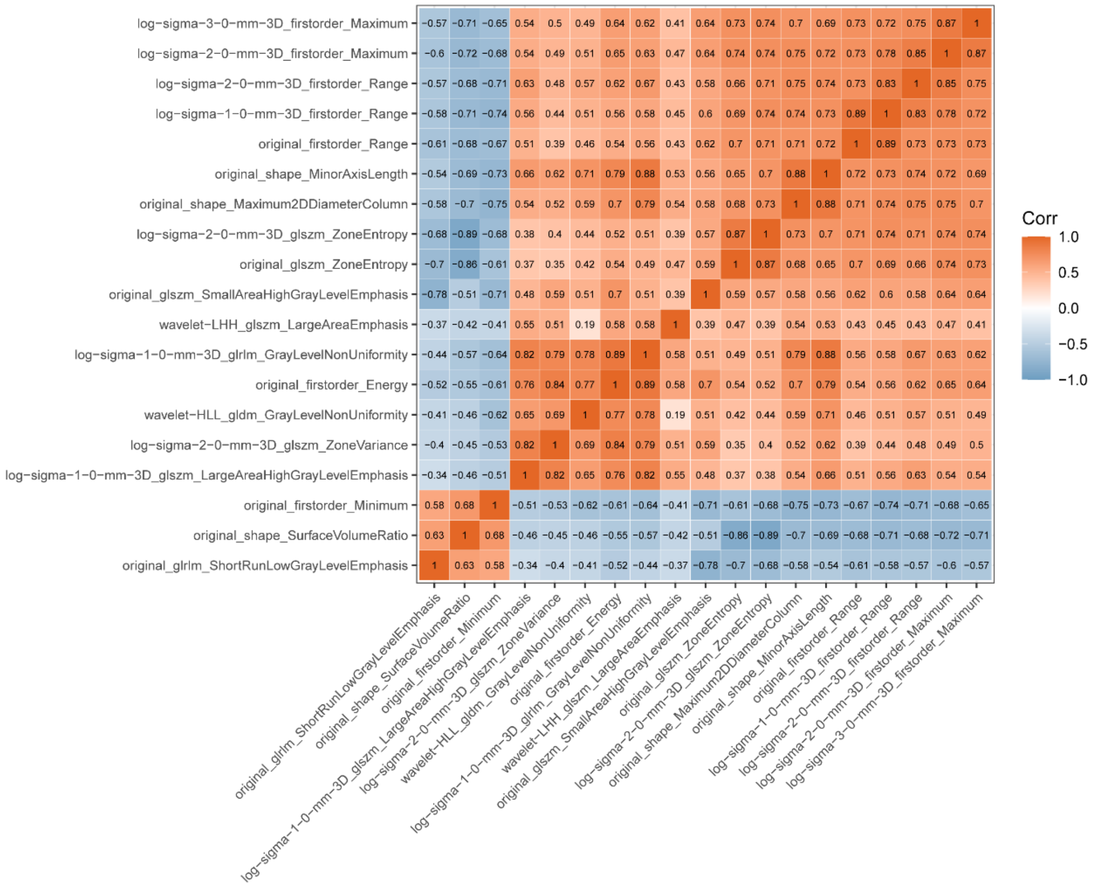

Figure S3. Correlation heat map of the selected 19 features. All the features' correlation coefficients are less than 0.9.

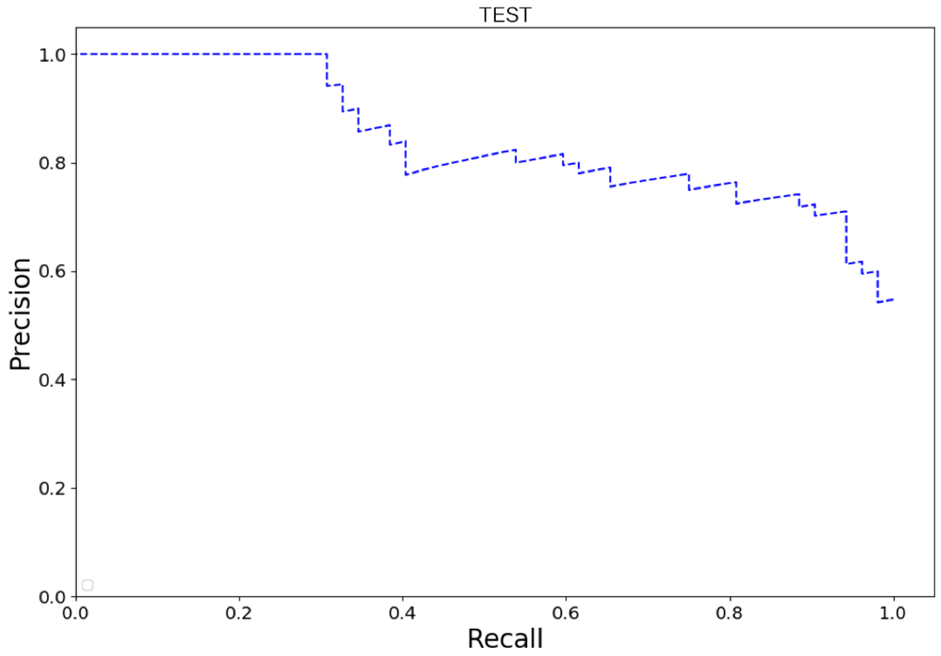

Figure S4. The precision-recall graph of LR radiomics model in the test set.

Supplement Tables

Table S1. Demographic and laboratory characteristics of patients with COVID-19 in the training and test cohort.

	**Training set（N=124）**			**Test set（N=64）**		
	**Improvement(n=70)**	**Aggravation(n=54)**	***P*-value**	**Improvement(n=41)**	**Aggravation(n=23)**	***P*-value**
**Age**	55.0 [49.2;65.8]	60.5 [56.0;67.0]	0.030	52.72 (5.64)	57.89 (10.01)	0.063
**Sex**			0.227			0.251
Male	34 (48.6%)	33 (61.1%)		7 (38.9)	12 (63.2)	
Female	36 (51.4%)	21 (38.9%)		11 (61.1)	7 (36.8)	
**Temperature,℃**	37.5 [36.9;38.2]	37.8 [37.0;38.2]	0.488	37.34 (0.73)	37.59 (0.71)	0.307
**Length of hospitalized**	21.6 (7.70)	23.1 (9.65)	0.352	16.78 (5.12)	18.89 (6.17)	0.265
**Symptom to discharged interval**	25.0 [22.0;31.8]	32.0 [27.0;38.0]	0.001	22.94 (4.90)	29.26 (4.90)	<0.001
**Complication**						
Angiocardiopathy	16 (22.9%)	17 (31.5%)	0.383	7 (17.1%)	9 (39.1%)	0.098
diabetes	14 (20.0%)	12 (22.2%)	0.937	3 (7.32%)	5 (21.7%)	0.124
hypertension	17 (24.3%)	19 (35.2%)	0.260	5 (12.2%)	8 (34.8%)	0.050
COPD	8 (11.4%)	10 (18.5%)	0.393	4 (9.76%)	5 (21.7%)	0.263
chronic liver disease	7 (10.0%)	3 (5.56%)	0.511	3 (7.32%)	4 (17.4%)	0.240
chronic kidney disease	3 (4.29%)	5 (9.26%)	0.293	4 (9.76%)	3 (13.0%)	0.695
**Symptom**						
fever	55 (78.6%)	47 (87.0%)	0.324	26 (63.4%)	16 (69.6%)	0.824
cough	41 (58.6%)	37 (69.8%)	0.275	9 (22.0%)	12 (52.2%)	0.028

muscular soreness	14 (20.0%)	17 (31.5%)	0.210	3 (7.32%)	5 (21.7%)	0.124
headache	5 (7.14%)	11 (20.8%)	0.051	3 (7.32%)	5 (21.7%)	0.124
diarrhea	10 (14.3%)	5 (9.26%)	0.566	3 (7.32%)	2 (8.70%)	1.000
**Laboratory**						
white blood cell, *10^9/L	4.78 [3.99;6.05]	6.06 [4.83;7.29]	0.003	5.33 (1.39)	5.94 (1.18)	0.068
Neutrophil, *10^9/L	3.07 [2.41;3.67]	4.15 [2.69;6.78]	0.001	3.51 [3.09;4.19]	4.51 [3.20;5.44]	0.058
Lymphocyte, *10^9/L	1.21 [0.89;1.55]	0.86 [0.54;1.27]	0.001	1.31 [1.04;1.52]	0.98 [0.52;1.38]	0.014
Hemoglobin, g/L	113 (23.8)	101 (16.2)	0.002	110 [95.0;127]	102 [96.5;113]	0.316
D-dimer, mg/L	1.06 [0.66;1.93]	4.54 [2.11;10.1]	<0.001	1.52 [1.01;6.84]	5.21 [2.78;7.46]	0.006
C-reactive protein, ml/L	12.2 [8.52;26.0]	44.1 [11.8;70.8]	<0.001	17.2 [8.46;19.6]	35.5 [15.7;56.1]	0.001
albumin, g/L	38.0 [34.3;41.8]	36.9 [33.9;40.8]	0.523	36.1 [34.0;38.9]	38.1 [34.0;41.2]	0.284
LDH, U/L	377 [294;442]	540 [446;708]	<0.001	357 (84.2)	486 (126)	<0.001

Data are expressed as mean [standard deviation], median [IQR], or n (%), where n is the total number of patients in each group. *p* values are from independent sample t-test or Mann-Whitney U test (continuous variable), or Chi-square test, or Fisher's exact test (categorical variable). $P < 0.05$ indicates that it is statistically significant. LDH: lactate dehydrogenase. IQR: interquartile range.

Logistic regression is a widely used interpretable algorithm and works well if a single decision boundary exists, with stable and satisfying performance in radiomic analysis. Of the selected features, gldm and glszm belong to texture feature, and glszm feature accounts for the largest proportion, which can quantify the gray level zones that are defined as the number of connected voxels that share the same gray level intensity in an image. Large Area Emphasis (LAE) of the glszm feature, which constitutes the largest proportion of the model, is a measure of the distribution of large area size zones, with a greater value indicative of larger size zones and more coarse textures. The first-order energy feature is a volume element representing the smallest unit of three-dimensional space division.

Table S2. Details of the 19 selected radiomics characteristics of the Logistic Regression (LR) model.

Feature	coefficient	relative_weight

wavelet-HLL_gldm_GrayLevelNonUniformity	1.0273	1
original_glszm_SmallAreaHighGrayLevelEmphasis	0.9339	0.9091
original_glszm_ZoneEntropy	0.9079	0.8838
log-sigma-2-0-mm-3D_firstorder_Maximum	0.3057	0.2976
wavelet-LHH_glszm_LargeAreaEmphasis	0.3002	0.2922
log-sigma-1-0-mm-3D_glszm_LargeAreaHighGrayLevelEmphasis	0.2451	0.2385
original_firstorder_Range	0.128	0.1246
original_glrlm_ShortRunLowGrayLevelEmphasis	0.0935	0.0911
log-sigma-3-0-mm-3D_firstorder_Maximum	0.0741	0.0721
log-sigma-1-0-mm-3D_glrlm_GrayLevelNonUniformity	0.0358	0.0349
log-sigma-2-0-mm-3D_glszm_ZoneVariance	0.0226	0.022
original_shape_MinorAxisLength	-0.0036	-0.0035
log-sigma-2-0-mm-3D_firstorder_Range	-0.1011	-0.0984
original_firstorder_Minimum	-0.1103	-0.1074
log-sigma-2-0-mm-3D_glszm_ZoneEntropy	-0.1325	-0.129
log-sigma-1-0-mm-3D_firstorder_Range	-0.1536	-0.1495
original_firstorder_Energy	-0.1549	-0.1507
original_shape_Maximum2DDiameterColumn	-0.3849	-0.3747
original_shape_SurfaceVolumeRatio	-0.7565	-0.7364

Feature values are named in three levels: first-level names indicate whether the filter is used, original indicates that the filter is not used; log: Laplacian of Gaussian; wavelet represents the wavelet transform. The second level is divided into three categories: first-order, shape, texture (glszm, gldm). The third level is the name of the specific features.

Table S3. Delong's test among the three kinds of models.

Model 1	Model 2	P-value
Radiomics model	Clinical model	0.698
Radiomics model	Combined model	0.714
Clinical model	Combined model	0.103

Table S4. Delong's test within the different models in the training set.

**Model1.ROC**	**Model2.ROC**	***P*-value**
Logistic Regression	Support Vector Machine	0.007
Logistic Regression	Decision Tree	0.015
Logistic Regression	Random Forest	0.779
Logistic Regression	Extreme Gradient Boosting	0.095
Support Vector Machine	Decision Tree	0.633
Support Vector Machine	Random Forest	0.020
Support Vector Machine	Extreme Gradient Boosting	0.472

Decision Tree	Random Forest	0.012
Decision Tree	Extreme Gradient Boosting	0.182
Random Forest	Extreme Gradient Boosting	0.037
